# A snapshot of patient experience of illness control after a hospital readmission in adults with chronic heart failure

**DOI:** 10.1186/s12912-023-01231-x

**Published:** 2023-03-21

**Authors:** Stephanie Turrise, Nina Hadley, Denise Phillips-Kuhn, Barbara Lutz, Seongkum Heo

**Affiliations:** 1 University of North Carolina, Wilmington, 601 S. College Road, Wilmington, NC 28403 USA; 2 Novant Health New Hanover Regional Medical Center, Wilmington, NC USA; 3 Mercer University, Macon, GA USA

**Keywords:** Heart failure, Hospitalization, Illness behavior, Self-management, Patient readmission

## Abstract

**Background:**

Approximately 6.5 million adults have chronic heart failure (HF), the number one cause of 30-day hospital readmission. Managing HF and its symptoms is critical for patients. Hospitalization may impact patients’ perceptions of illness control, which can affect illness management. However, how hospital readmissions are perceived as related to one’s ability to control their HF and its symptoms has not been examined.

**Objective:**

The purpose was to explore the experiences of people with HF in managing their illness (i.e., illness control), understand their perceptions of illness control after recent hospital readmission, and clarify the concept of illness control in people with chronic HF.

**Methods:**

A qualitative approach, applied thematic analysis was employed. Purposive sampling was used to identify participants. Semi-structured interviews were conducted in 10 participants’ homes. Ongoing, concurrent, and comparative data analysis was used with ATLASti© data management software.

**Results:**

Two themes were identified, strategies to control HF and barriers to controlling HF. Strategies to control HF included four subthemes: managing dietary intake and medications; self- advocacy; monitoring symptoms; and support. Barriers to control also had four subthemes: healthcare systems issues; health care professional relationships and interactions; personal characteristics; and knowledge deficits.

**Conclusion:**

People use many different strategies to control HF. Control comes from both within and outside of the individual. The desire to control HF and its symptoms was evident, but implementing strategies is challenging and takes time, experience, and trial and error. Individuals did not view readmission negatively but as necessary to help them control their symptoms.

## Introduction and background

Heart failure (HF) is a progressive condition that has become a significant public health problem, and the prevalence increases with age [1]. According to the American Heart Association 6.5 million Americans 20 years and older suffer from this chronic condition with prevalence projected to increase to over 8 million by 2030 [2]. HF is one of five diagnoses responsible for 20% of the United States (US) national healthcare costs and is one of the most expensive conditions billed to Medicare [3]. One important cause of the high costs is the high rates of hospitalization. According to the United States Department of Health and Human Services Healthy People 2030, the metric “reduce heart failure hospitalizations” is getting worse rather than better [4] and few interventions have positively affected hospital readmission rates [5].

Controlling HF requires that individuals adhere to a complex treatment plan of dietary changes, take medications as prescribed, and monitor signs and symptoms, including daily weight, edema, dyspnea, and activity tolerance [6]. In an integrative systematic review, many factors were identified as antecedents to hospital readmission, including non-adherence to medications, inadequate knowledge, and misperceptions about disease management [7]. Regardless of how well people understand, adhere and manage their illness, readmission may not be prevented entirely. In this case, readmission itself may impact perceptions, which can affect their symptom management. However, how these readmissions are perceived in relationship to one’s ability to control their HF and its symptoms has not been examined.

In previous studies, some researchers reported counterintuitive findings regarding an individual’s belief that they can control their illness as a risk factor for readmission and the impact of perceived control on medication adherence. For example, in one study individuals who indicated that they had higher levels of personal control of their illness were more likely to experience medication non-adherence, an unexpected finding [8]. This was consistent with Ross and colleagues [9], who reported higher adherence to medications in individuals with low personal control beliefs in subjects with hypertension. Additionally, people who believed their treatment was not controlling their HF had higher all-cause readmission rates [8]. These findings imply that perceived control may impact self-care and hospitalization. In practice, nurses encourage personal control and self-care, but some people with chronic illnesses have difficulty adhering to recommendations for various reasons. Furthermore, patients expressed frustration because they believed they *were* controlling their illness but were still being hospitalized [8]. Many people with HF are admitted to the hospital frequently, which may influence perceptions of illness control.

Illness management in people with HF, is poor [10] and is one important reason for delaying seeking treatment and hospitalization [11, 12]. On the other hand, hospitalization itself may impact an individuals’ perceptions of illness control, impacting HF self-care. Thus, better understanding perceptions of illness control related to hospitalization and HF self-care is needed. However, not many researchers have examined this from patients’ perspectives. The purpose of this study was to explore the experiences of illness control in people with HF and understand their perceptions of how illness control and hospital readmission affected their perceptions and symptom control.

## Study aims

This study aimed to explore individuals experiences in controlling their illness (i.e., illness control), understand their perceptions of illness control after a recent hospital readmission, and gain clarity around the concept of illness control in people with chronic HF. Findings from this study will help to understand how individuals define illness control and determine if and how their perceptions of control impact their illness management and self-care.

## Theoretical framework

**Fig. 1:**
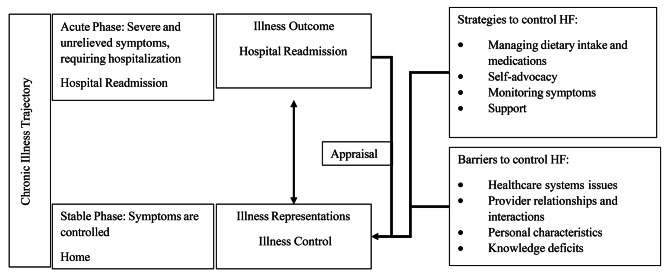
Figure depicting study theoretical models

Two theories guided the study and provided a broad framework to understand how study participants define illness control in HF and how it may change when a hospital readmission occurs (Fig. 1).

Illness control comes from the theory the Common Sense Model of Illness Representations (CSM) [13]. The CSM is a self-regulatory framework developed to describe health and illness behaviors [14]. Illness control is one’s anticipated and perceived responsiveness to self-treatment and expert intervention [14]. Illness control is one of five dimensions that illness representations are formed upon [14] and contributes to illness self-management and adherence, impacting illness outcomes in chronic illness [15–18].

Corbin and Strauss’ Chronic Illness Trajectory Model [19, 20], a grounded theory that describes the process of managing distinct phases in the trajectory of a chronic illness, also guided this study. The illness trajectory, delineated by phases (see Table 1), and the work to manage it are shaped by events, such as hospitalizations. In this study, individuals are in the transition from the acute phase of the trajectory, where they have severe or unrelieved symptoms or complications of their illness that required a recent re-hospitalization for stabilization, to the stable phase, where the illness course and symptoms are under control.

**Table 1:** Corbin and Strauss Phases, Definitions and Goals of Management

Trajectory Phase	Phase Definition	Phase Management Goals
Pretrajectory	Prior to the onset of the illness	Prevention of chronic illness
Trajectory Onset	Illness signs and symptoms are present	Diagnosis and forming trajectory projection
Crisis	Life threatening episode that requires emergency or critical care	Removing the threat
Acute	Illness is active and requires management in the hospital	Control the illness and resume normal activities of daily living (ADLs)
Stable	Course of illness and/or symptoms controlled by regimen	Maintenance of illness stability and ADLs
Unstable	Course of illness and/or symptoms are not controlled by regimen, but does not require hospitalization, being managed at home	Return to stable phase
Downward	Progressively deteriorating with an increase in disability and symptoms	Adapting to the disability and managing symptoms
Dying	The immediate time period, hours to weeks, preceding death	Peaceful death

Corbin, 1998; Corbin & Strauss, 1991; Reed & Corner, 2013

The concepts of illness control, illness management, and self-care at first glance, may seem similar. However, they are different and have important distinctions and roles in HF care outcomes. Illness control is ones’ belief about how an illness will respond to treatment and is a factor in behavior according to the CSM. Illness management then is the work of managing an illness or the behaviors undertaken and is influenced by beliefs. Lastly, HF self-care, as defined by Riegel and colleagues [21] is a “naturalistic decision-making process that influences actions that maintain physiologic stability, facilitate the perception of symptoms, and direct the management of those symptoms” (p. 226). Based on these theories, the illness control beliefs are derived from cognitive and emotional processing, which drives the actions and decision-making that are foundational to understanding the broader concept of HF self-care. Consequently, knowing what people with HF believe about illness control is essential.

## Methods

Qualitative research follows an inductive approach, using individual experiences to understand a phenomenon. In this study, the qualitative approach of applied thematic analysis [22] was used. Applied thematic analysis allows for a rich and in-depth description and interpretation of an experience or phenomenon while staying close to the data [22, 23]. Applied thematic analysis [22] is a rigorous approach that uses a “set of procedures designed to identify and examine themes from textual data in a way that is transparent and credible” (p. 15). The focus of the analysis in this study was on describing and interpreting the participants’ experiences with HF control by identifying codes in the narrative and transforming them into key themes.

### Procedures

Institutional Review Board approvals were obtained from the recruitment site and the University of North Carolina Wilmington. Potential participants were identified by review of the electronic health record and by nursing staff on the cardiac unit. The electronic health record identifies patients that have been readmitted with a symbol. Further review was necessary to validate that the index hospitalization and readmission were due to HF. Once potential participants were identified, a member of the research team, who was also a staff member at the recruitment site, visited the patient and family, explained the study, provided an information sheet, and explored their interest in participating. If they were interested, their contact information was given to the PI, who called within the week to arrange a visit for the interview at a location of the patient’s choice, which in all instances was their residence. This setting was ideal because it allowed the context, relations, and interactions within their environment to be assessed [24] and is important in qualitative research.

### Sampling

A purposive sampling strategy was used to select participants. Participants were adults who could speak, read and write English, gave informed consent, and had been readmitted to the hospital for HF exacerbation within 30 days of an index hospitalization for HF and were being discharged home. Individuals were excluded if they were terminally ill or being discharged home on hospice, or had dementia. Two participants asked that their caregiver be a part of the interview. The first five participants were predominantly male (80%) and had HF for a long time, ranging from 2 to 10 years. Consequently, we purposefully recruited women and participants with a more recent diagnosis, specifically between newly diagnosed and one year of living with HF.

Participants were recruited from an inpatient medical telemetry unit at a level two trauma medical center in southeastern North Carolina. A total of 11 individuals were recruited. However, only 10 participated in interviews, with one being lost to follow-up after discharge.

Themes began to repeat with the sixth interview suggesting data saturation. At that point, recruitment focused on purposeful female recruitment as the first six participants were primarily male (5 out of 6) and had their HF for a year or longer. Four additional interviews were conducted to verify preliminary themes and ensure no new information emerged.

### Interviews

All interviews were conducted by the PI (ST) between June 2015 and February 2016. Interviews were conducted using open-ended questions and probes as needed. Interview questions were designed to explore the participants’ perspectives about their illness control while limiting researcher influence. Interviews were audio recorded and transcribed by a transcription service. The PI validated transcripts for accuracy before analysis. The ongoing, concurrent thematic analysis allowed for more focused, purposeful sampling so that participants were recruited with characteristics or experiences that helped address questions or gaps in the data. Interviews ranged in length between 25 and 75 minutes, with an average length of 43 minutes. Pseudonyms are used throughout to maintain the confidentiality of participants.

### Data analysis

As transcriptions were completed, data were analyzed in two ways. Three researchers on the team (DK, NH, and ST) reviewed and coded the transcripts looking for commonalities and differences among the data within and across interviews [22]. Data analysis consisted of coding by identifying similar phrases, patterns, themes, sequences, and key features. Each author did the coding independently. In the second step, the coding was reviewed, and any discrepancies or differences were discussed until a consensus was reached. Once this was completed, the codes were examined to determine if some broader categories or themes could be derived from the data and compared to existing knowledge and theories. An expert qualitative researcher (BL) met with the PI to review the coding structure and related data and assisted with conceptual interpretations. The themes began to repeat with the sixth interview. Four additional interviews were conducted to verify preliminary themes and ensure no additional information emerged. The transcripts were entered into ATLASti©, a qualitative data analysis software program. This software helped with data management and coding.

The study’s rigor was maintained using such strategies as purposeful sampling, accurate transcription of the data, and establishing rapport with the participants and their family members to enhance honesty and openness during the interviews. Qualitative data collection procedures were recorded and reported in detail to strengthen transferability. The interview guide with probe questions was developed by two of the authors (ST and BL) and was revised based on feedback from qualitative experts at a qualitative research “boot camp” and from the other authors to ensure that the guide covered key topic areas, was theoretically driven, and enhanced credibility. Two of the authors (ST and DK) have extensive experience working with people who have HF, one author is an expert in qualitative research (BL), and three authors have experience disseminating research findings (ST, BL, DK) which further enhances credibility.

Triangulation was used during data analysis by having all team members conduct within-case and cross-case analyses. As themes or categories were identified, they were compared within and across interviews to explore the data for similarities and differences in participants’ experiences. Transferability was achieved using journals, completed after each interview by the PI to keep field notes about the environmental cues, feelings, thoughts, and participant non-verbals related to the interview itself. Memoing was also used throughout data analysis to track ideas about emerging concepts and emerging themes and the relationships among them. The data collection and analysis process is iterative; therefore, these strategies helped the researchers identify gaps in understanding and develop additional focus questions for later interviews.

## Results

Participants ranged in age from 53 to over 90 years old and represented six counties surrounding the medical center, 5 of which are designated rural according to the 2014 census. There were two females and eight males, and they reported having had their HF diagnosis between 2 months and 10 years. Other demographic data are reported in Table 2.

**Table 2:** Study Sample Demographic Characteristics

Variable	*n*	%
**Gender**		
Male	8	80
Female	2	20
**Race**		
Black/African American	5	50
White	4	40
Native American	1	10
**Marital Status**		
Married	3	30
Divorced	3	30
Separated	0	0
Widowed	4	40
Never married	0	
**Lives**		
Alone	5	50
With spouse	2	20
With family	3	30
**Work Status**		
Retired	6	
Unable to work	6*	Some answered as
Disabled	0	both retired and
Working	0	unable to work
Unemployed	0	
Never worked	0	
**Education level**		
Less than high school graduate	2	20
High school graduate	3	30
Community college education	3	30
Baccalaureate graduate	1	10
Graduate school	1	10
**Income level**		
Less than $25,000/year	6	60
Between $25,000 and $50,000/year	0	0
Between $50,000 and $75,000/year	0	0
Between $75,000 and $100,000/year	1	10
More than $100,000/year	0	0
Declined to answer	3	30
**Years with HF diagnosis**		
Less than 1 year	6	60
1–4 years	2	20
5–10 years	2	20
**Number of Comorbidities**		
1–3	5	50
4–6	4	40
> 6	1	10
**Number of Prescribed Medications**		
1–5	4	40
6–10	3	30
> 10	3	30

### Patient experience of illness control

Two themes were identified: strategies used to control HF and barriers to controlling HF. Strategies to control HF included four subthemes: managing dietary intake and medications; self-advocacy; monitoring symptoms; and support. Barriers to control had four subthemes: healthcare systems issues, health care professional (HCP) relationships and interactions, personal characteristics, and knowledge deficits.

**Strategies to control HF.** The strategies to control HF were what people did or identified that helped them control or manage their HF. They reflected aspects of illness management, including managing dietary intake and medications, self-advocacy, monitoring symptoms, and support.

***Managing dietary intake and medications*** One way participants controlled their HF was by managing their dietary and medicinal intake. All interviewees said they had received education on dietary restrictions, specifically “salt” or sodium intake. However, many participants stated they were uncertain about the amount of sodium they could ingest or where to look for sodium content in foods. Others indicated that they were not always compliant with restrictions or used incorrect strategies. For example, one interviewee knew sodium was essential to monitor but used commercially prepared sauce for dinner that evening. All participants *knew* sodium was something to avoid in their diets, and most said they did not add salt to their food. However, some acknowledged intentional noncompliance. One participant said, “Well, uh, when I go out to eat, certain things are, um, full of salt” (Reilly).

Individuals had a superficial understanding of the connection between sodium and fluid retention and the subsequent effects on their hearts. “Salt makes a lot of fluid, and I’ve laid off my salt….I just put a little bit in my rice when I cook my rice, but I don’t add it after it’s cooked no more” (John) and “But you know, they have me on some, um, on a kinda strict diet… no way the salt do not agree with me” (Ralph). Another unclear dietary area for participants was whether they needed to restrict fluid intake or how to balance fluid intake with diuresis. “They said that I was dehydrated, getting dehydrated. I had to drink more fluids and stuff….but I-I don’t know why I’m still gaining” (John) and another said, “Sometimes though we pull off a little too much [fluid] and that’ll make his blood pressure drop. So we have to be careful how we do it” (Logan).

All participants recognized medications as necessary for HF control. However, views on who was responsible for the medications varied. For example, some individuals felt that taking medications was something they controlled themselves. Others saw family members or significant others who managed the medications as responsible. In contrast, others suggested it was solely up to the HCP who made prescribing decisions as being in total control of this aspect of care. Participants focused on medications and their role in controlling HF, particularly when compared to behavioral and lifestyle modifications. “I said I’m not taking it. And I missed a day and a half and felt better. Then I thought. I said, nah, I’ve got to take it because I don’t want the fluid back on me, so I went back to taking it” (John).

***Self-advocacy*** Many participants expressed the importance of self-advocacy or having a family member advocate on their behalf when they were too ill. They wanted to be viewed and respected as experts on their bodies and symptoms. “No. I know my body. I know how it felt… I know the difference” (Amos).

This theme persisted across time living with HF, from those who had only been diagnosed for a month to those with longstanding HF. However, those with longstanding HF appeared to have more proficiency and confidence in determining the cause of their symptoms and were more vocal about it with their HCPs.

*“Like I said, be proactive. Learn all about your disease you can. Educate yourself. Ask the tough questions. If you don’t think the doctor is doing like he wanted to or wassupposed to or think he should do, ask him why. I used to be scared to ask the doctor anything, but now I’m not because if you don’t, I mean, how you gonna find out?“* (Karl).

***Monitoring symptoms*** All participants discussed the “constant monitoring,“ “vigilance,“ and “constant checking” of their symptoms. Common symptoms that they tracked included their breathing ability, insomnia, restlessness, anxiety, and fatigue or, as described in their words, “sluggishness” (Amos), “washed out, I felt like I’d been through a washing machine, a wringer” (Reilly) and “not just tired, exhausted” (Bernice). The seriousness or severity of symptoms guided actions and care-seeking. For example, one individual said, “when you can’t breathe, it is critical” while another knew that the symptoms were severe enough to warrant a call to 911 rather than a call to the physician’s office. One participant noted that he utilized the symptoms to decide whether to seek care, and if so, where, either at the doctor’s office or the hospital, stating that all of the services that he would need would be centrally located at the hospital and that this may be the better option. Some individuals expressed having warning signs that their HF was worsening, “…I can tell when it’s fixing to start, I can tell” (Karl), while another said, “Since he’s had it so many times, we know when the symptoms are coming” (Logan). In contrast, others described a more sudden and rapid onset “It came back fast, it built up on him real fast” (Joy), and another individual also said, “just all of a sudden, just out of nowhere, it started again” (Jim). Multiple comorbid conditions also played a role in monitoring and interpreting symptoms. Individuals that had other health problems, particularly lung problems such as asthma and chronic obstructive pulmonary disease had greater difficulty discerning the cause and the appropriate response. For example, one interviewee said, “I thought it was my lungs that were the problem but turns out it was my heart” (Logan).

***Support*** Support was a key strategy to successfully managing and responding to HF. Support, expressed as intrinsic and extrinsic to the individual, came from many sources, including spiritual, family, significant others, HCPs, and others with HF. Individuals used this support as a source of strength, “I thank God for our family, the unit because you can pull off each other’s strength” (Logan) and was viewed as guiding HCPs in caring for people with HF. Family members encourage healthy behaviors, such as quitting smoking, avoiding sodium and taking medications, and aiding with daily living activities by moving in temporarily or offering their own homes as a place to recover. Others mentioned seeking the advice of friends who have HF to find out what worked for them, their experiences, and who they recommended for care, both individual HCPs and hospitals. One participant expressed that while he did not know anyone with HF, he thought this would have been an asset as he navigated the illness.

**Barriers to controlling HF.** Barriers were things individuals identified that they had to overcome or prevented them from controlling or managing their HF and were rooted in the health system, relationships with HCPs, knowledge deficits, and personal characteristics.

***Healthcare systems issues*** The healthcare system presented many challenges, including sharing of information or coordination of care among facilities, such as the veteran’s administration and the local hospital, as well as problems within each facility. One participant said, “… just before it was time to go, to leave, there were all these different medicines. No one had a grip on what medicines that I was on. I mean, it was a mess” (Jim). Another individual felt that they were not getting what they needed from the hospital they were going to,

“*I believe in going to the doctor, trying to find out what was wrong. And I felt like, okay, after two times in the hospital, and I’m still feeling the same way, I’m still sick, I’m still tired, and, I’m like, you know, this is crazy. What do I do? I’m not getting better. I felt like the system failed me*” (Amos).

Frustration with the system and thinking they were following directions and doing what they were told but still not feeling better were commonly expressed. Additionally, individuals said that they understood that the goal of hospitals, insurers, and HCPs providers is for patients to stay out of the hospital. They also did not want to be hospitalized. However, their experiences and the reality of HF are that it is progressive, with periods of exacerbations and remissions requiring hospitalization.

***Challenges in provider relationships and interactions*** Healthcare professionals inadvertently communicating that a person can *always* control their HF and stay out of the hospital is not entirely true. These messages may cause a delay in care-seeking and a more severe presentation once individuals reach care. Every participant described some delay in seeking care. Understanding each individual’s rationale for delays is essential in care management. The other message people reported receiving is to go to the emergency room (ER) if they do not feel well. “You could call your doctor. You can’t get to talk to them, you know, —so you have to wait a day or two. You’re gonna have to wait to get in. They tell you, “If it’s important, go to the ER,“ so I went to the ER.“ Another commonly discussed problem was the uncertainty or lack of awareness that HF was the problem at the initial hospitalization, but once they were readmitted, all the stops came out. On index admission, Jim said, “They didn’t, they didn’t mention anything to us about heart failure, did they? … The readmission is when they actually began to run these tests, and they found out for sure what it was,“ while another, Reilly said “The first time, when I came home, I didn’t have any nurse. Now they’ve got me with the visiting nurse. She comes twice a week …”. Many participants discussed how the readmission was a “relief.“ They knew they needed to be hospitalized to feel better and get the help they needed for symptom control. John said, “he (the cardiologist) said, ‘Well, I’d rather you be in the hospital,‘ and I said, ‘I’d rather be there. This is one time that I need help really’.“

All participants described challenges in their relationships with their HCPs. Mistrust of HCPs, poor communication between HCPs and patients and among HCPs, lack of advocacy by nurses, and missed opportunities were the negative experiences mentioned by participants about their interactions and relationships with doctors, nurses, therapists, and other providers such as emergency medical services. One participant felt that if the HCPs who are experts could not identify worsening HF, how could he? “And I was getting bigger and bigger and bigger. And I kept asking I said, ‘What are we gonna do about this?’” (Karl). Another individual spoke about the severity of his scrotal swelling and difficulty walking, yet, his concerns were not addressed. However, despite these factors, positive relationships enabled them to overcome barriers. For example, one participant described the HCPs practice as “the factory” and was cynical about the cookie-cutter feeling he got at the office. Still, he continued going there because he liked and trusted his HCP.

***Personal characteristics and beliefs*** Some personal characteristics and beliefs impacted the interactions with the healthcare system, and in turn an individuals’ illness control. One participant stated that she felt that the hospital administrators and staff just “do not care about patients” and will “just get you well and then go on and send you home” (Bernice), and others felt that there was hesitancy to get “specialists” (Logan) or “cardiologists” (Jim) involved. Individuals recognized that they were a barrier to managing their HF, alluding that their habits and ways are ingrained and difficult to change, such as smoking or dietary habits. One participant said, “Let me tell you something, I’m 72 years old. I don’t intend to change anything at my age now, and I don’t” (Joy), while another advised that those with HF should “Do as I say, not as I do” (John).

***Knowledge deficits*** Finally, knowledge deficits and misunderstandings were also barriers to managing HF. Some examples included a knowledge deficit on the correct time to weigh, misunderstanding the proper dietary restrictions, and misinterpretation of symptoms, mainly when there were multiple comorbidities, which contributed to the increase in illness management complexity. One individual said about daily weights, “I used to, wasn’t doing it.… but now I weigh two or three times a day” (John), while another said that after the readmission, “I do that now once I get out the bed morning time…” (Ralph). When asked about a HF diet, one individual stated, “I have cut back on eating … more to lower calories” (Joy), confusing the diet for her diabetes with the diet for HF. Another participant said, “… well, the second time I was in ‘cuz they, uh, said I had, uh, what is it, COPD? And that’s part of the problem, my breathing and whatnot” (Logan) while others failed to understand the chronic nature of HF, “… my heart is good, uh I still got eight more years to go” (Reilly), while another said, “I’ll be glad when I get away from— (the water pills) and those big ole horse pills (potassium supplement)” (Logan).

## Illness control after hospital readmission

In addition to the strategies for managing HF and barriers to HF control, information about whom people with HF felt was responsible for control after experiencing hospitalization was discovered. The concept of control, who was in control, and how the HF was controlled, seemed to vary across participants. This finding follows the CSM theory, in which factors such as patient experiences, cultural considerations, and individual beliefs about the dimensions, such as timeline and control, drive the development of illness representations and direct self-regulation and behavior. Many individuals, while they knew that HF required them to adopt behavioral changes related to diet, fluid, and symptom monitoring and medications, relied upon others, such as significant others or HCPs to help regulate them, particularly the medications. Healthcare professionals reinforced illness management and self-care, but patients felt limited in what they could do. “… the doctor has told us that it’s something that we can manage but we can’t manage it but so far” (Logan).

Another individual expressed understanding that medicines control HF but felt that the HCPs ultimately direct this area of care, and it can be a point of confusion “…just before it was time to go, to leave, was all these different medicines. No one had a grip on what medicines that I was on. I mean, it was a mess” (Jim).

Another participant echoed the sentiments of medicine controlling their HF and other comorbid conditions when he stated, “That’s here what keep me livin’, so I’ll take it. I might as well take it” (Ralph). In addition, illness control was related to individuals’ clinical characteristics, that is, time from diagnosis of HF or how long they had lived with HF. As might be expected, those who participated in this study and had HF for a longer period were more expert on their symptoms, what had to be done, and how to do it, compared to those who were newly diagnosed or had their HF for a shorter period.

Overall, many participants worked hard to control their illness, did what their HCPs recommended, and still felt they needed more support and guidance. When they ran into trouble, they sought care, usually at the ER, because they had reached a critical point, such as being unable to breathe. Others believed they understood what they needed to do to control their illness but were not motivated to implement necessary changes and admittedly recognized this. Knowledge about the illness, personal symptoms, and what to do improved with time, meaning the longer they had HF, the better they were at controlling, managing and recognizing worsening HF symptoms.

## Discussion

To our knowledge, this is the first study to examine the patient experience of illness control after discharge from a hospital readmission for HF. There were similarities across individual experiences and significant differences in patients’ perceptions of illness control. Two themes were identified. The first theme, strategies used to control HF consists of four subthemes. The subthemes show that after discharge, patients tried to control their illness by managing diet, medication, and symptoms and advocate for themselves with support from others. However, they faced several barriers to illness control, which was the second theme. The second theme also consisted of four subthemes. The subthemes show that barriers came from several sources, such as healthcare systems, HCPs, and their own, such as their negative perceptions and lack of knowledge. These findings present essential targets of interventions to improve illness control, such as more support from HCPs and significant others and more effective communication between patients and HCPs to enhance effective strategies for the management of diet, medication, and symptoms.

Participants reported receiving information on medications, diet, daily weighing, and their importance and verbalized the necessity of these illness management strategies. However, it was clear that their understanding was incomplete and often inaccurate, and they had difficulty implementing the strategies once they were at home. In addition, even when they knew what they should be doing, some could not because they could not afford or access the equipment and tools (scale, medication, cardiac rehabilitation) and the right foods (e.g., fresh produce, non-processed foods). It was also evident that individuals knew to record the data, such as daily weights, but were not sure what else to do with that other than to “bring it to the doctor” or for those using telehealth or other nursing services such as home health, that the “nurses are looking at that.” In other words, they frequently did not know how to act on that data because they were not informed, empowered, or confident or did not understand the relationship between weight, fluid retention, and HF illness management. Individuals with an understanding seemed to question their decision-making and so poignantly stated that if the HCPs had difficulty identifying and managing their HF, how could they be expected to do so? They had difficulty identifying the source of symptoms, particularly when they had comorbidities. These findings align with the propositions in the Situation-Specific Theory of Heart Failure Self-care [21, 25] that state symptom recognition is essential to successful self-care management, that people with more knowledge, skill, experience, and compatible values have better self-care, and that confidence moderates and mediates the relationship between self-care and outcomes.

Additionally, beyond self-confidence or the belief that something can be done is self-efficacy, which according to Bandura [26] is the belief in one’s capacity to execute certain behaviors which affect thinking, feeling, motivation and action. In the HF literature, these terms are often used interchangeably, but conceptual differences may impact illness control. It is possible that those low in self-efficacy rely on others via social support to assist with HF self-care, impacting their beliefs about illness control and who exerts the control.

Having a voice and being heard about symptoms and other aspects of their HF was essential to participants, and many stated that either they or their family members advocated for their needs. While this may go against the notion that they do not manage well, it reveals the tension and difficulties between being motivated to self-manage, thinking they are self-managing, and being appropriately equipped and prepared to do so. Dunbar and colleagues [27] suggest that family support influences the HF patient’s behavioral characteristics and directly affects self-care. Additionally, Jaarsma and colleagues [28] state that using self-management skills in everyday life is insufficient. These skills must be practiced in context to manage HF successfully. Corbin and Strauss [19] also note that the work of managing chronic illness takes place in people’s homes, not hospitals and rehabilitation centers and that the work is done not just by the patient but in conjunction with family and caregivers. Nurses can intervene and implement strategies to help patients and families improve self-management and illness control by providing guidance and education where the work occurs in the home. Nurses can empower patients and caregivers to ask questions and become a collaborator in care rather than relying on others to manage and control it. According to Brashers, Goldsmith, and Hsieh [29], this information-seeking and facilitation is a form of social support consistent with our findings. This informational support assists patients with identifying symptoms or possible treatments and can come from family, friends, or HCPs. Heo and colleagues [30] also found that more social support was significantly associated with higher levels of perceived control.

However, in this study, participants wanted their HCPs to appreciate their “self-expertise” or being the expert on their bodies. This is consistent with the collaborative care or partnership model, in which professionals are experts about the disease, but patients are experts about their own lives [31]. The two need to be leveraged to improve self-care. Utilizing and maximizing these resources appears to be essential in making the journey from novice to expert and controlling chronic HF. This also has policy implications for home-based services eligibility and how these services are reimbursed.

A significant theme related to the concept of illness control and self-care emerged. Over time, individuals described acquired expertise, a self-knowing, an accumulation of knowledge that they acquired both about their symptoms and how to control them as well as what works best for them. For example, individuals with longstanding HF knew what to do, were more likely to question their providers, and self-advocate for things they felt they needed. This is consistent with the findings of Clark and colleagues [32] who report in a systematic review that effective interventions helped individuals understand the nature and complexities of managing HF, which could accelerate the “learning oneself” timeline. However, in addition to the complexity of care, Dickson and Riegel [33] suggest that the skills for self-managing HF and controlling symptoms evolve over time with practice. Furthermore, understanding that HF is a progressive illness that may not be controllable or that as HF progresses, symptoms may change and strategies for managing it may also need to change. This idea is one of the key components of Corbin and Strauss’ Trajectory framework [19], where they state, “chronic conditions have a course that varies and changes over time” (p.156). Heart failure is no different.

## Limitations and future research

The current study aimed to explore chronic HF patients’ experiences in controlling their illness (i.e., illness control), understand their perceptions of illness control after a recent HF hospital readmission and gain clarity around the concept of illness control. Qualitative research is hypothesis-generating and not intended to be generalizable; thus, this is not a limit so much as an opportunity to deeply and richly explore an individual’s experience. This sample, which allowed data saturation and a wide representation of age, time living with HF, and race was representative of the region the recruitment site serves but may not represent different experiences of those living in other regions, countries, or types of communities, such as a larger city. The sample was also skewed male, despite significant efforts to recruit female participants. Despite these limitations this study contributes essential information on illness control from those living with chronic HF. However, future research, quantitative and mixed methods, can further assess and explore differences between individuals living in rural vs. urban areas, different segments of socioeconomic status, educational level, and sex. Additionally, quantitative research may be able to identify predictors of good and poor illness control and hospital readmission.

## Conclusion

Controlling HF is attempted using many different avenues by patients. This control comes from both within and outside of the individual and includes things such as medications and self-care behaviors, but it requires supportive relationships from family, significant others and HCPs. For many individuals, the desire to control the condition and its symptoms is evident but putting the pieces into place is challenging and takes time, experience, and trial and error to determine the best course of action. Individuals did not view readmission negatively; they deemed a return to the hospital as their only option due to how they felt. This suggests that individuals require assistance, coaching, or guidance in the places and spaces they return to after hospitalization.

Determining what the individual wants or how engaged and activated they are to participate in the necessary changes is critical and should shape the treatment plan and conversations on managing their HF. One way of overcoming these barriers was having a trusted HCP. Strengthening these relationships may be a key strategy for helping individuals improve their self-management skills and abilities. Finally, examining communication with individuals and families about their HF, its controllability, and how it is managed. Confusing or mixed messages and focusing on the problems rather than the strengths may impair patient engagement in self-care.

## Data Availability

The data that support the findings of this study are available from the corresponding author ST upon reasonable request. The data are not publicly available because this is qualitative data and the information contained in the interviews could compromise research participant privacy/confidentiality and goes against consent.
